# Declines in benthic macroinvertebrate community metrics and microphytobenthic biomass in an estuarine lake following enrichment by hippo dung

**DOI:** 10.1038/srep37359

**Published:** 2016-11-17

**Authors:** Jessica Dawson, Deena Pillay, Peter Jean Roberts, Renzo Perissinotto

**Affiliations:** 1 Marine Research Institute, University of Cape Town, Biological Sciences department, Cape Town, 7701, South Africa; 2 DST/NRF Research Chair in Shallow Water Ecosystems, Nelson Mandela Metropolitan University, Port Elizabeth, 6031, South Africa

## Abstract

Hippos transfer massive quantities of trophic resources from terrestrial to aquatic ecosystems through defecation. The ramifications of the latter for the functioning of benthic ecosystems are unknown, but are dependent ultimately on rates of utilisation relative to inputs. Low input and high utilisation can strengthen bottom-up pathways and enhance consumer biomass and abundance. However, if inputs exceed utilisation rates, dung can accumulate, leading to a decline in water quality, with important repercussions for resident assemblages. Here, we quantify the consequences of hippo dung inputs on benthic assemblages in an estuarine lake in South Africa. The system supports over a thousand hippos, and during recent drought periods (extending over a decade), hippo dung has been observed to form mats over benthic habitats. Enrichment of plots using exclusion/inclusion cages with dung at naturally occurring concentrations indicated a decline in benthic chl-*a* by roughly 50% and macrofaunal abundance, biomass and richness by up to 76, 56 and 27% respectively. Our findings suggest that persistent inputs of hippo dung can act as an important stressor of benthic systems, leading ultimately to a loss of productivity. Accumulation of hippo dung over benthic habitats is therefore an important mechanism by which hippos indirectly structure aquatic ecosystems.

Hippos (*Hippopotamus amphibius*) are iconic components of aquatic ecosystems in Africa. However, despite dominating gravimetrically and ecologically, hippos rank amongst the most understudied faunistic components on the continent^1^. The roles hippos play in physically engineering aquatic ecosystems, and the feedbacks transmitted to sympatric species is a particular gap in knowledge, though these have frequently been alluded to in the literature^2^,^3^,^4^,^5^. The direct transfer of trophic resources from terrestrial to aquatic ecosystems is one of the most significant functions provided by hippos across landscapes in which they occur^3^,^6^,^7^,^8^. By consuming large quantities of terrestrial grasses by night and excreting this material into aquatic systems by day^3^,^5^,^9^,^10^, hippos translocate trophic resources across terrestrial-aquatic boundaries at rates and magnitudes that are unlikely to be replicated by other natural phenomena. In the Mara River, for example, it has been estimated that individual hippos defecate approximately 8.7 kg of grasses (wet weight) into the river per day, and that the entire population transfers 36 tonnes of grass into the river daily^10^.

Hippo-induced trophic transfers have the potential to alter aquatic assemblages at several organisational levels^1^,^2^,^3^,^11^. Essentially though, the latter can occur through two broad functionally distinct pathways, depending on frequencies and magnitudes of transfers. Firstly, transfers can subsidize recipient ecosystems, resulting in bottom-up interactions being strengthened and consumer biomass and/or abundance increasing^12^. However, if rates and magnitudes of transfers exceed rates of utilisation, then transfers can accumulate, leading to deteriorating water quality and physiological stresses on communities^1^. Local hydrodynamics is a particularly important determinant of the rate at which trophic transfers accumulate, with low flow likely to enhance dung accumulation and retention times^6^. Aquatic systems that experience low hydrodynamic forcing and have high densities of hippos may be particularly prone to dung accumulation and associated biotic and abiotic feedbacks. The broad objective of this paper was to quantify the consequences of persistent hippo dung inputs and accumulation on benthic assemblages in the St Lucia Estuary, South Africa, using *in situ* mesocosm experiments. The estuary is home to over a thousand hippos, which have been estimated to transfer roughly 2000 tonnes (dry mass) of dung into the system per year^2^. The estuary has historically undergone cyclical drought phases, with the most recent lasting roughly a decade. During droughts, the mouth of the system can close off from the sea, resulting in hypersaline conditions developing, particularly in the upper reaches, where salinities in excess of 200 psu have been recorded^13^,^14^,^15^. In addition, significant portions of the system can dry out, resulting in the estuary becoming fragmented into smaller water bodies^16^,^17^. During the latest drought period, hippo dung has regularly been observed to form dense mats on the benthos, and has frequently been recorded in benthic grab samples, particularly in areas where hippos are common. The latter formed the core rationale underlying the central objective of the study. The general approach adopted involved experimental enrichment of plots with hippo dung at concentrations recorded naturally at sites in the Narrows (Fig. 1), where roughly 50% of the hippo population resides^2^. Based on our observations of dung forming dense mats (up to 1 cm in thickness) over the benthos in the Narrows, we hypothesized that enrichment of the benthos with hippo dung would generate biologically significant shifts in benthic communities, particularly by depressing macro-invertebrate community metrics and microphytobenthic biomass.

## Methods

**Figure 1:**
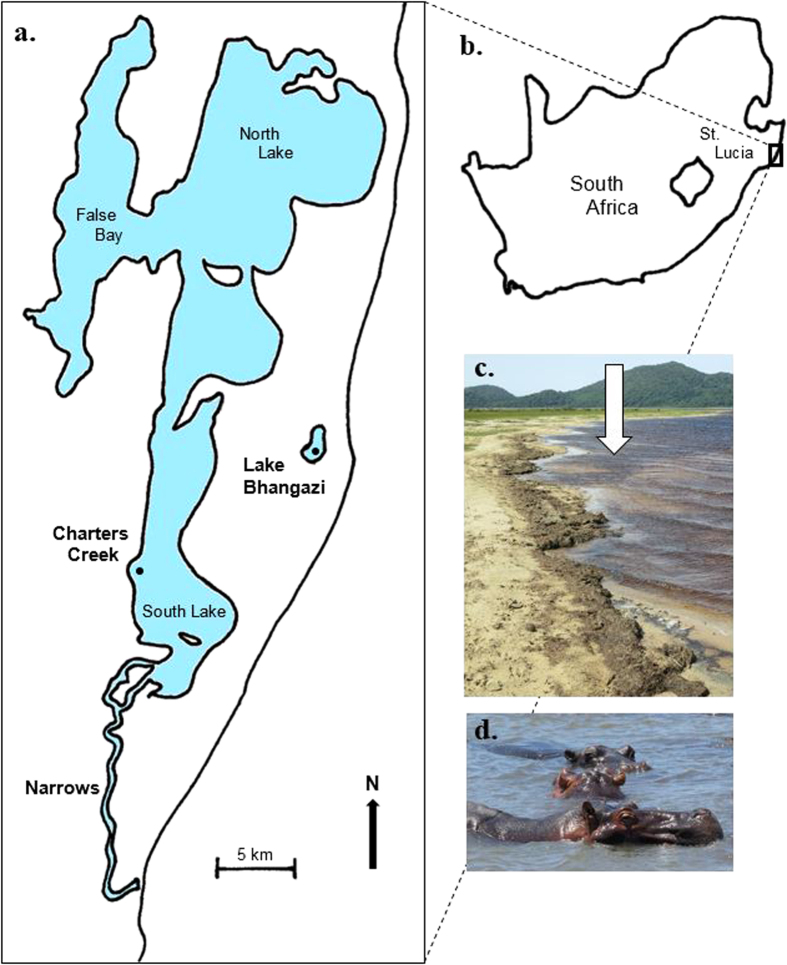
(**a**) Map of the St Lucia Estuary, showing its location in South Africa (**b**). (**c**) Accumulation of dung (indicated by arrow) excreted by resident hippos (**d**).

Manipulative field experiments were undertaken at two sites in Charter’s Creek in the St Lucia Estuary (27°52′S and 28°24′S and 32°21′E and 32°34′E, Fig. 1). The estuary is the largest estuarine system in Africa^18^ and forms part of the iSimangaliso Wetland Park, which was recognised as a world heritage site in 1999, in appreciation of its biodiversity and regional importance^13^,^19^. The system is composed of three shallow (0.5 m) interconnected lakes that discharge into the Indian Ocean *via* a deeper (2 m) channel referred to as the Narrows (Fig. 1). The Narrows is dominated by mud/silt (<63 μm, 75% of total), while sediment at Charter’s Creek, in South Lake, is dominated by medium (500–250 μm) and fine sand (250–125 μm), contributing 39.8% and 46.2% to total sediment composition^17^. Turbidity within the system is highly variable, with values ranging from 1 NTU (Nephelometric Turbidity Units) to 951 NTU, with highest values generally recorded at the Narrows and Charter’s Creek^17^. Similarly, background nutrient concentrations are highly variable, with Dissolved Inorganic Nitrogen (DIN) ranging between 0.001 and 770 μM and Dissolved Inorganic Phosphorus (DIP) between 0.0001 and 15.14 μM^17^.

Historically, hippos were distributed throughout the system, but drought conditions and anthropogenic manipulation have resulted in a greater displacement of hippos towards the south. While hippos are still present in the Lakes, they occur there in lower numbers while the Narrows now contains roughly 50% of the St Lucia hippo population^2^. The sites chosen to conduct the experiments are infrequently utilised by hippos and thus offered a relatively safe and undisturbed location to quantify responses of benthic systems to hippo dung enrichment. At the time of conducting the experiment (October to November 2014), the mouth of the system had been closed off from the Indian Ocean since 2002, with a brief opening between March and August 2007^14^. Under these conditions, water flow in the lakes is very limited, though mixing through wind is observed^20^.

Ten inclusion/exclusion cages (length and width = 50 cm, height = 1 m) were randomly interspersed (2–3 m apart) at each of the two experimental sites (150 m apart, water depth = 40–50 cm), with two treatments being assigned (dung inclusion and dung exclusion; n = 5 per site) at each site. Each cage consisted of a frame comprising four rods (2 cm diameter) that were hammered 30 cm into the sediment and surrounded by 3 mm mesh. Tops of cages were uncovered and protruded 10 cm above the surface waters. After installation, cages remained unmanipulated for two days. Fresh hippo dung (within 24 hours of being voided) was collected along hippo paths at Lake Bhangazi, on the Eastern Shores of the estuary (Fig. 1). Dung from 3–5 individual middens was homogenised and roughly 300 ml added to dung inclusion cages, once per week for six consecutive weeks. During weekly dung additions, there were no indications of previously added dung being present, which probably reflects rapid breakdown through wind mixing and microorganism activity. The volume of dung added to inclusion cages was based on a mean volume obtained from benthic grab samples (n = 2 per site, area = 0.026 m^2^, depth = 20 cm) collected at nine sites in the Narrows, and scaled to the area of each experimental cage. These sites covered a total distance of 2–3 km, and were inhabited by three hippo pods (between 15 to 30 hippos per pod). All grab samples were collected within a 150 m radius of hippo pods. Replicate grab samples were emptied into buckets and sieved (500 μm and 2000 μm) within 8 hours, and retained dung emptied into a measuring jar. No dung was added to dung exclusion cages.

Sediment cores for assessing responses of microphytobenthic biomass (as chl-*a*; n = 2 per cage, diameter = 2 cm, depth = 1 cm) and macrofaunal assemblages (n = 2, diameter = 10 cm, depth = 15 cm) were collected at the termination of the experiment. Chl-*a* samples were refrigerated in 30 ml of acetone (90%) for 48 h, followed by fluorometric determination of chl-*a* concentrations (Turner Designs Trilogy fluorometer). All macrofauna cores were passed through 500 μm and 2000 μm sieves respectively, and retained material preserved in an ethanol (70%) and Rose Bengal solution. Macrofauna were identified to the lowest possible taxonomic level, enumerated and the biomass of each taxon determined per sample (Mettler ToledoMX5, precision = 1 μg).

PRIMER v6.1 was used for all multivariate analyses based on unstandardized and transformed (log (x + 1)) abundance data. Spatial variability in macrofaunal community structure was visually assessed using CAP (canonical analyses of principal co-ordinates) and statistically assessed using PERMANOVA (permutational analysis of variance), based on Bray-Curtis similarity matrices. For PERMANOVA analyses, a nested hierarchical design was employed with site as the highest spatial factor, within which treatments (dung exclusion/inclusion) were nested. ABC (abundance biomass comparison) curves were constructed on aggregated community data per treatment at each site using the DOMINANCE function, in order to determine the influence of dung enrichment on ranked species biomass versus abundance. Macrofaunal community descriptors (total abundance, biomass and species richness) were calculated using the DIVERSE function. SIMPER (similarity percentages) was used to identify species that cumulatively contributed 90% to the dissimilarity between treatments at each site. Nested ANOVA (analysis of variance) was used to test the effects of site and dung treatments on benthic chl-*a* levels and macrofaunal community descriptors. The assumptions required for parametric testing were assessed using Q-Q plots for normality and Bartlett Tests for homogeneity of variances. Data were transformed where required prior to parametric testing. All univariate statistical tests were conducted in the data analysis platform R.

## Results

**Figure 2:**
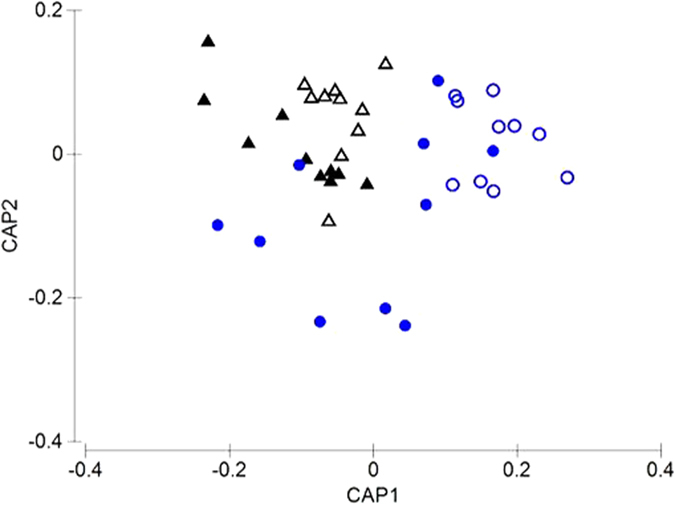
Canonical analysis of principal co-ordinates (CAP) plot showing spatial variation in macrofaunal community structure in Site 1 dung inclusions (▴) and exclusions (△), Site 2 dung inclusions (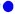) and exclusions (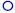).

At the community level, enrichment of plots with hippo dung caused significant shifts in the structure of macrofaunal assemblages relative to exclusions (PERMANOVA pseudo F_2,39_ = 2.64; p = 0.003). Site differences in community structure were statistically insignificant (PERMANOVA pseudo F_1,39_ = 1.57; p = 0.312). CAP plots visually confirmed the results produced by PERMANOVA, which showed a clear distinction in macrobenthic assemblages between dung exclusion and inclusion plots (Fig. 2) at both sites.

**Figure 3:**
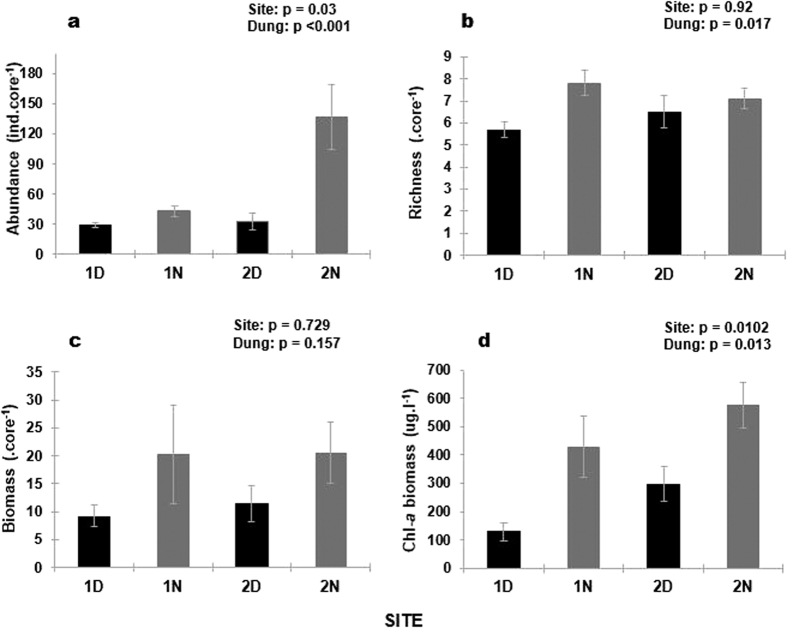
Spatial variation in mean (±s.e.m.) (**a**): macrofaunal abundance, (**b**) species richness, (**c**): biomass and (**d**): microalgal biomass (as chl-*a*) between dung exclusion and inclusion plots. Numbers in site name = site number; D = dung addition, N = dung exclusion.

Dung addition generally resulted in a depression of macrofaunal community descriptors (Fig. 3). Macrofaunal abundances were significantly different between inclusion and exclusion treatments (Nested ANOVA; F_2,32_ = 18.61; p < 0.001), being reduced by 32 and 76% at Sites 1 and 2 respectively. Site differences in macrofaunal abundance were also statistically significant (Nested ANOVA; F_1,32_ = 5.15; p = 0.03), generally being greater in Site 2 than Site 1. Macrofaunal richness was significantly reduced in dung inclusion treatments (Nested ANOVA; F_2,32_ = 4.64; p = 0.017), though this was most evident in Site 1, where dung addition resulted in a decline in richness by roughly 27%. Dung addition also reduced macrofaunal biomass by 44 and 56% at the two sites, but the latter was not statistically supported due to high variance in the data (Nested ANOVA; F_2,32_ = 1.963; p = 0.157). Lastly, chl-*a* biomass was roughly halved at both sites following addition of dung (Fig 3; Nested ANOVA; F_2,31_ = 4.99; p = 0.013). Chl-*a* levels were greater at Site 2 than Site 1 (Nested ANOVA; F_1,31_ = 7.488; p = 0.01).

In terms of individual species responses, SIMPER identified seven and five taxa that cumulatively accounted for 90% of the dissimilarity between treatments at both sites. Of the dominant species found at Site 1, six decreased in abundance following addition of dung, while abundance of all dominant species declined at Site 2 (Table 1). All six dominant species in terms of biomass decreased with dung addition at Site 1, while at Site 2 six of the seven dominant species decreased following dung addition (Table 1).

**Figure 4:**
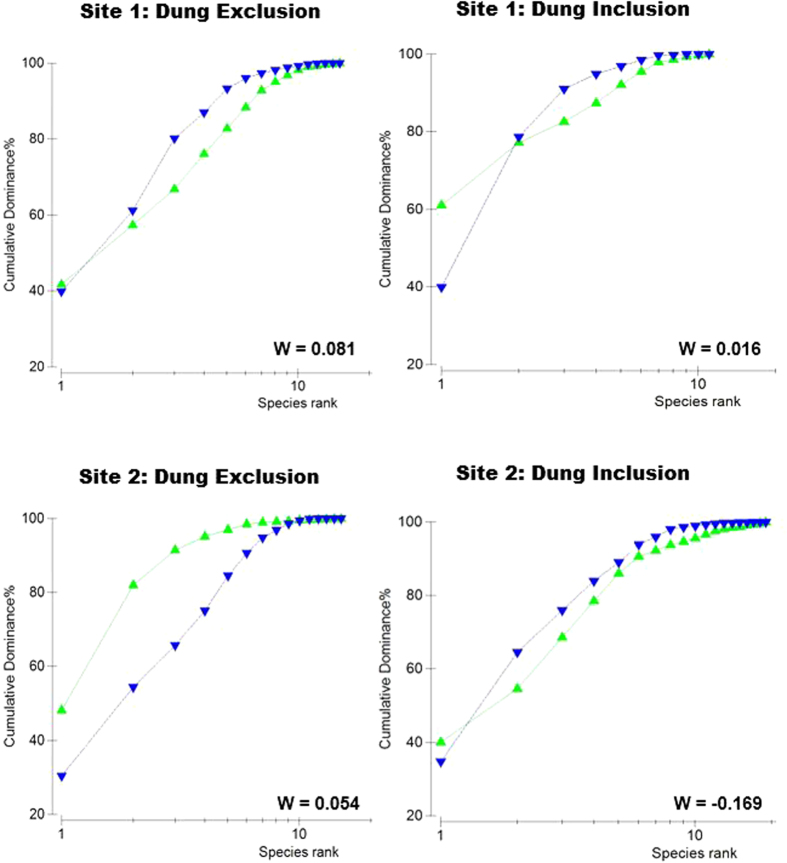
Cumulative dominance plots showing ranked species abundance (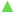)and biomass ( 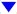
**) for macrofaunal assemblages in dung inclusion and exclusion treatments at Sites 1 & 2.**

Cumulative abundance-biomass plots showed interesting effects of hippo dung addition on benthic communities (Fig. 4). In Site 1, dung addition, caused abundance of dominant species to increase relative to biomass, resulting in a decrease in W-statistic from dung exclusions (W = 0.081) to inclusions (W = 0.016). In effect, dung addition caused a shift in communities to r –selection. In Site 2, the opposite occurred, with W-statistics increasing from dung exclusions (W = −0.169) to inclusions (W = 0.054).

## Discussion

**Figure 5:**
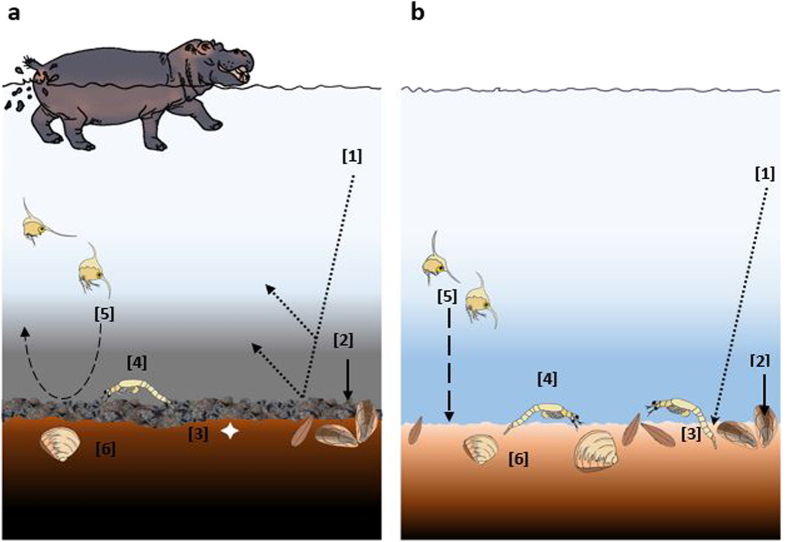
Schematic overview of differences in macrobenthic communities in the presence (**a**) and absence (**b**) of hippo dung and the hypothesised mechanisms by which the latter structures benthic macrofaunal assemblages. At high inputs, dung can [1] reduce light penetration by increasing turbidity, leading to a decline in benthic microalgal biomass; [2] increase levels of suspended organic matter, which can inhibit filter-feeding organisms; [3] enhance sediment abrasion leading to interference with surface-dwelling organisms; [4] act as a barrier for recruitment or [5] a negative settlement cue for pelagic taxa; [6] increase anoxia and hydrogen sulphide levels, which stresses organisms.

Our results indicate that persistent inputs and accumulation of hippo dung at levels recorded in the Narrows may play an important ecological role in structuring benthic ecosystems in the St Lucia Estuary, principally by depressing benthic community metrics and microphytobenthic biomass. Such effects are likely driven by various mechanisms operating interactively or individually, as indicated in Fig. 5. Declines in benthic microalgal biomass are probably driven by dung shading the benthos, causing a reduction in incident light available for photosynthesis. While there is no direct evidence in the literature pointing to hippo dung causing shading of benthic environments, the latter can be inferred from studies reporting a reduction in light penetration and productivity in response to elevated suspended organic matter levels^21^,^22^. The latter could negatively feedback to benthic consumers, given the importance of benthic microalgae as trophic resources for benthic organisms^23^, and could thereby account for the reductions in macrofaunal abundance and that of dominant taxa, following experimental dung enrichment. Studies have also highlighted the susceptibility of filter-feeding taxa to suspended material in the water column, as the latter interferes with filtration ability^24^. The above may explain the reduction in contributions of filter feeding bivalves (*Brachidontes virgiliae* and *Meretrix morphina*) to assemblage composition in the presence of hippo dung. Oxygen depletion associated with decomposition of dung, or the blooms of algae caused through dung inputs^1^,^25^ could impose physiological stresses for benthic organisms, thereby leading to declines of sensitive taxa. Dung may also physically abrade the benthos, potentially eroding the upper sediment layers along with surface-associated organisms. Lastly, the presence of hippo dung may limit recruitment of benthic taxa with pelagic larval stages either by (i) functioning as a barrier that restricts movement of recruits across the pelagic-benthic interface, or (ii) creating conditions that function as negative settlement cues (low oxygen and high ammonia levels) for recruits^26^. The reduction in abundance of crab zoea in the presence of dung, especially at Site 2, is suggestive of hippo dung being capable of impacting juvenile stages of benthic macrofauna.

Functional traits of organisms have been highlighted as important predictors of community responses to environmental stressors^27^, and are therefore likely to be equally important in determining the magnitude of hippo dung effects on benthic assemblages. It could be argued that since hippo dung accumulates at the sediment-water interface, organisms living on or close to the sediment surface would be most susceptible to the effects of dung accumulation. It is also generally accepted that surface-associated organisms are largely smaller than deeper dwelling infauna and endobenthic taxa. Our data provide some support for a hypothesis that smaller, surface-associated taxa would be more susceptible to dung inputs. In our study, the greatest impact of dung addition at the community level (Fig. 2) and for macrofaunal abundance (Fig. 3) occurred at Site 2, which was dominated by two surface-associated taxa viz. the mysid *Mesopodopsis africana* and crab zoea. These two taxa also demonstrated the greatest response magnitudes to dung enrichment. Further support for the hypothesis that smaller, surface associated taxa would be most susceptible to hippo dung comes from abundance-biomass comparisons, which showed that dung addition at Site 2 caused a decline in dominance of taxa with low biomass (viz. *M. africana* and crab zoea).

Conditions that enhance accumulation and persistence of hippo dung over benthic substrates are likely to strengthen the effects on benthic assemblages observed in our study. Two important conditions impacting the latter would be rates of flow within aquatic ecosystems and volumes of dung defecated, with low flow coupled with high dung volumes likely to enhance dung accumulation^1^. Closed pond/pool ecosystems for example, which are known to be inhabited by hippos^1^,^2^, are likely to be most susceptible to dung retention, given the largely impermeable nature of their boundaries relative to lotic systems. Anthropogenically-induced reductions in freshwater inputs into aquatic ecosystems can lead to significant flow reductions^28^, while droughts, which are a feature of arid and semi-arid climates, are capable of amplifying flow declines^13^. The St Lucia Estuary is a system that typifies the latter points, as damming, water abstraction and artificial closure of the Mfolozi River, which is one of the largest tributaries flowing into the system, have led to an estimated loss of 54% of original flow^20^,^29^. During the latest drought, the mouth of the system remained closed-off from the sea for roughly 10 years, with close to 80% of the lakes evaporating and fragmenting^16^. At one stage, close to 90% of the lakes was lost due to drought and the artificial manipulation of the system^20^. Such conditions are likely to facilitate retention and concentration of hippo dung, which, based on our findings, would depress benthic community metrics and microalgal biomass over-and-above background stressors. Therefore, under drought conditions, hippo dung accumulation may function as an important secondary stressor for benthic ecosystems, which would operate in parallel with the primary effects of hypersalinity, habitat desiccation and recruitment limitation through prolonged mouth closure^13^.

Taken collectively, our results expand current understanding of the role played by an iconic African megaherbivore in structuring aquatic ecosystems. More specifically, we demonstrate that persistent accumulation of hippo dung can function as a key determinant of benthic assemblage structure. Based on the levels of dung used in our experiment, the drought conditions and anthropogenic changes experienced by the St Lucia Estuary, our results indicate that accumulation of hippo dung can lead to reductions in benthic community metrics and microalgal biomass. However, the possibility does exist that at reduced dung inputs and conditions that facilitate diffusive transport, hippo dung may induce stimulatory bottom-up effects on benthic assemblages. Our results indicate that the accumulation of hippo dung over benthic habitats should therefore be considered as an important mechanism by which hippos indirectly engineer aquatic ecosystems in Africa.

## Additional Information

**How to cite this article**: Dawson, J. *et al.* Declines in benthic macroinvertebrate community metrics and microphytobenthic biomass in an estuarine lake following enrichment by hippo dung. *Sci. Rep.* **6**, 37359; doi: 10.1038/srep37359 (2016).

**Publisher’s note:** Springer Nature remains neutral with regard to jurisdictional claims in published maps and institutional affiliations.

## Figures and Tables

**Table 1:** Macrofaunal species cumulatively accounting for community dissimilarity (90%) among treatments at each site based on abundance and biomass data.

Dominant taxa (abundance data)	Site 1		Dominant taxa (abundance data)	Site 2	
	Dung	No Dung		Dung	No Dung
	Average Abundance			Average Abundance	
*Polydora* sp. (P)	17.90	18.00	*Mesopodopsis africana* (M)	12.90	66.10
Crab zoea	1.00	6.70	Crab zoea	13.10	49.90
*Brachidontes virgiliae* (B)	0.70	4.00	*Polydora* sp. (P)	4.70	12.90
*Ceratonereis keiskama* (P)	4.70	4.10	*Ceratonereis keiskama* (P)	4.50	5.20
*Cyathura estuaria* (I)	1.60	2.90	Cumacea (C)	2.40	2.50
*Mesopodopsis africana* (M)	1.40	2.40			
Cumacea (C)	1.40	1.90			
**Dominant taxa (biomass data)**	**Average Biomass**		**Dominant taxa (biomass data)**	**Average Abundance**	
*Cyathura estuaria* (I)	3.68	4.31	*Mesopodopsis africana* (M)	4.00	6.24
*Polydora* sp. (P)	3.57	3.85	Crustacean zoea	0.92	4.93
*Meretrix morphina* (B)	0.00	8.09	*Cyathura estuaria* (I)	3.42	0.86
*Ceratonereis keiskama* (P)	1.14	1.28	*Ceratonereis keiskama* (P)	1.31	1.94
*Brachidontes virgiliae* (B)	0.19	1.39	*Dendronereis arborifera*	0.55	1.94
*Dendronereis arborifera* (P)	0.14	0.55	*Polydora* sp. (P)	0.58	2.32
			*Meretrix morphina* (B)	0.00	1.26

P: polychaete, B: bivalve, I: isopod, M: mysid.
